# Influenza Vaccine Effectiveness against Influenza A-Associated Outpatient and Emergency-Department-Attended Influenza-like Illness during the Delayed 2022–2023 Season in Beijing, China

**DOI:** 10.3390/vaccines12101124

**Published:** 2024-09-30

**Authors:** Li Zhang, Guilan Lu, Chunna Ma, Jiaojiao Zhang, Jia Li, Wei Duan, Jiaxin Ma, Weixian Shi, Yingying Wang, Ying Sun, Daitao Zhang, Quanyi Wang, Da Huo

**Affiliations:** 1 Institute for Infectious Disease and Endemic Disease Control, Beijing Center for Disease Prevention and Control, No.16 He Pingli Middle St, Dongcheng District, Beijing 100013, China; zhanlgi@bjcdc.org (L.Z.);; 2 Beijing Research Center for Respiratory Infectious Diseases, No.16 He Pingli Middle St, Dongcheng District, Beijing 100013, China; 3 School of Public Health, Capital Medical University, 10 Xitoutiao You’anmenwai St, Fengtai District, Beijing 100069, China

**Keywords:** influenza, vaccine effectiveness, test-negative design, China

## Abstract

Background: During the 2022–2023 influenza season, the influenza activities in most regions of China were postponed, including Beijing. The unusually delayed influenza epidemic posed a challenge to the effectiveness of the influenza vaccine. Methods: Using the test-negative design, we evaluated influenza vaccine effectiveness (VE) during the 2022–2023 influenza season against influenza A-associated outpatient and emergency-department-attended influenza-like illness (ILI) in Beijing, China, from 9 January to 30 April 2023. Results: The analysis included 8301 medically attended ILI patients, of which 1342 (46.2%) had influenza A(H1N1)pdm09, 1554 (53.4%) had influenza A(H3N2), and 11 (0.4%) had co-infection of the two viruses. VE against influenza A-associated ILI patients was 23.2% (95% CI: −6.5% to 44.6%) overall, and 23.1%, 9.9%, and 33.8% among children aged 6 months to 17 years, adults aged 18–59 years, and adults aged ≥60 years, respectively. VE against influenza A(H1N1)pdm09 and against influenza A(H3N2) were 36.2% (95% CI: −1.9% to 60.1%) and 9.5% (95% CI: −34.1% to 39.0%), respectively. VE of the group with vaccination intervals of 14–90 days (70.1%, 95% CI: −145.4 to 96.4) was higher than that of the groups with a vaccination interval of 90–149 days (18.7%, 95% CI: −42.4% to 53.6%) and ≥150 days (21.2%, 95% CI: −18.8% to 47.7%). Conclusions: A moderate VE against influenza A(H1N1)pdm09 and a low VE against influenza A(H3N2) were observed in Beijing during the 2022–2023 influenza season, a season characterized with a delayed and high-intensity influenza epidemic. VE appears to be better within three months after vaccination. Our findings indicate a potential need for the optimization of vaccination policies and underscore the importance of continuous monitoring of influenza to enhance vaccines and optimizing vaccination timing.

## 1. Introduction

From 2020 to 2022, seasonal influenza circulation was suppressed by restriction measures due to the coronavirus disease 2019 (COVID-19) pandemic in China, including in Beijing. After historically low influenza activity during the COVID-19 pandemic, a significant delayed intense influenza activity was observed during the 2022–2023 influenza season in Beijing following the relaxation of COVID-19 restriction measures in early January 2023 through China [1], with the dominant influenza virus subtypes of A(H1N1)09pdm and A(H3N2). This out-of-season epidemic pattern was typical in northern China during this season, while it was inconsistent with the epidemic trend globally [2]. According to the surveillance reports from North American and European countries, the influenza activity in these regions in the 2022–2023 influenza season was relatively earlier than that before COVID-19 [3,4,5,6]. Studies indicated that the protection effect of influenza vaccine would decline over time and be significantly attenuated after six months of vaccination [7,8]. As the large-scale influenza vaccination in Beijing in the 2022–2023 influenza season was carried out between September and November, which was about 3 to 5 months ahead of the onset of the influenza epidemic, it provided us with an opportunity to explore the impact of the delayed influenza epidemic on the vaccine protection effect.

In addition to the variation of vaccine-induced antibody titers over time, the degree of antigenic matching between vaccine strains and the circulating influenza viruses is another important factor influencing the effectiveness of influenza vaccines in a given season [9]. During the 2022–2023 vaccination campaign, trivalent or quadrivalent inactivated influenza vaccines (IIV) were used in Beijing, following the recommended composition of egg-based influenza virus vaccines for the Northern Hemisphere by World Health Organization (WHO) [10]. Reports of the Chinese National Influenza Center (CNIC) showed that from 3 October 2022 to 30 April 2023, 1.8% of influenza A(H1N1)09pdm strains and 49.9% of influenza A(H3N2) strains had low response to vaccine strains A/Victoria/2570/2019 (H1N1)pdm09-like virus and A/Darwin/9/2021 (H3N2)-like virus, respectively [11]. This indicated the necessity to characterize the genetic features of this influenza season.

In this study, we evaluated 2022–2023 influenza vaccine effectiveness (VE) against influenza A-associated outpatient and emergency-department-attended influenza-like illness (ILI) in Beijing, and we assessed how VE changes by time intervals between vaccination and illness onset. In addition, we describe the genetic features of representative strains that circulated in Beijing during the season.

## 2. Methods

### 2.1. Study Design, Participants, and Laboratory Detection

The test-negative case–control study design was adopted to assess VE against influenza A-associated influenza-like illness (ILI) in outpatient and emergency department visits, details of which have been described previously [12,13]. In summary, epidemiological information and specimens of participants were collected through the influenza virological surveillance system managed by the Beijing Center for Disease Prevention and Control (BJCDC), which consisted of 23 sentinel hospitals and 17 collaborating laboratories in the 2022–2023 influenza season. Under this system, ten to twenty pharyngeal swab specimens of medically attended ILI cases were collected by healthcare workers in outpatient settings (including internal medicine, pediatric clinic, and fever clinic) and emergency department in each sentinel hospital per week. We defined ILI as having a fever ≥38.0 °C with cough or sore throat. Samples of ILI patients who visited within 3 days after the onset were priority collected. The specimens were transported to collaborating laboratories in viral transport medium at 4 °C for nucleic acid detection of influenza virus and SARS-CoV-2 by reverse transcription polymerase chain reaction (RT-PCR). SARS-CoV-2-positive cases (including co-infections with influenza), as well as a small number of influenza B-positive cases, were excluded from the analysis.

Sanger sequencing of the viral haemagglutinin (HA) gene was undertaken on a subset of original specimens to access the contribution of genetic clades to VE estimates. A total of 35 influenza A(H1N1)09pdm strains and 35 influenza A(H3N2) strains isolated during the period from February to April 2023 were randomly selected and sequenced. Viral RNA was extracted using Cq-Ex DNA/RNA Kit (TIANLONG, Xian, China) following the manufacturer’s instructions. For strains, reverse transcription and amplification of the HA gene were carried out as described previously [14,15]. Then, PCR products were sequenced by ABI Prism 3130xl automated sequencer (Applied Biosystems, Foster City, CA, USA). Nucleotide sequences were assembled and then aligned by MEGA software (ver. 5.0). The maximum likelihood trees were constructed using the Hasegawa–Kishino–Yano model (Gamma distributed (G)) 1000 bootstrap replications.

### 2.2. Data Collection

Given the possible impact of the COVID-19 control measures on people’s medical-seeking behavior, this study selected the period from 9 January to 30 April 2023 after the relaxation of the COVID-19 measures as the research time period. Epidemiological information of sampled cases was collected by healthcare workers in the sentinel hospitals using a standardized electronic questionnaire, which included demographic characteristics (age, sex, occupation), underlying medical conditions, clinical information (onset date, visiting date, symptoms), and the interval between the onset and the swab collection time. Medical conditions included asthma; tuberculosis; pulmonary fibrosis; chronic tracheitis or bronchitis; emphysema; chronic obstructive pulmonary disease; diabetes; tumors; and hematologic, cardiovascular, cerebrovascular, renal, hepatic, neurological, and immune system conditions. Staff of collaborating laboratories were required to collect and integrate questionnaire data from sentinel hospitals and sent datasets to BJCDC.

Influenza vaccination information of sampled cases was obtained from the Beijing Management System of Information for the Immunization Program. According to the recommended immunization schedule of 2022–2023 in China [16], one dose of 0.5 mL IIV was used in people aged 9 years and above. For children aged 6–35 months and 36 months to 8 years, if it was the first time receiving the influenza vaccine, 2 doses of 0.25 mL or 0.5 mL IIV should be provided, respectively, with an interval of ≥4 weeks; if 1 or more doses of influenza vaccine had been received in the previous influenza seasons, one dose of 0.25 mL or 0.5 mL IIV should be provided to children of these two age groups, respectively. Patients who received one or two doses of IIV in accordance with the vaccination guideline 14 or more days before symptom onset were regarded as vaccinated. Patients vaccinated within 14 days before symptom onset were considered unvaccinated and excluded.

### 2.3. Statistical Analysis

Demographic and clinical characteristics by cases and vaccination status were described using counts and percentages. Vaccination status across groups were compared by chi-squared tests. Unadjusted and adjusted VE were calculated as (1 − unadjusted or adjusted odds ratios) × 100% using univariable and multivariable logistic regression models, respectively, by comparing the odds of influenza vaccination between test-positive cases verses test-negative controls. Multivariable logistic regression models were adjusted for potential confounding factors including age, sex, comorbidity, interval (≤3 days, >3 days) between illness onset and specimen collection, and calendar week of illness onset. Age and calendar week as continuous variables were modeled as natural cubic spline variables. The 95% confidence intervals (CI) for VE were calculated as (1 − CI_OR_) × 100%, where CI_OR_ is the 95% CI of the odds ratio estimates.

We estimated general VE against influenza A type and by influenza A(H1N1)pdm09 and A(H3N2) subtypes, stratified by age group (0.5–17 years, 18–59 years and ≥60 years) and by time interval between vaccination and ILI onset (14–89 days, 90–149 days and ≥150 days) where the sample size allowed. We also tested for potential effect modification by the prior (2021–2022) season’s influenza vaccine using indicator variable analysis. Since the influenza vaccine was provided for people over the age of 6 months, eligible participants for the study of previous vaccination were restricted to those aged ≥2 years in 2022–2023 with valid data for influenza vaccine receipt both in 2022–2023 and 2021–2022. Sensitivity analysis excluding participants with interval between ILI onset and swabbing >3 days was conducted to account for the possible bias of VE estimation caused by false negatives due to untimely sampling.

Questionnaire data were entered using EpiData software (version 3.1; The EpiData Association, Odense, Denmark), and data were analyzed using R version 4.0.3 (R Foundation for Statistical Computing, Vienna, Austria) software. All statistical tests were two-sided, with *p* < 0.05 considered statistically significant. The study was approved by the institutional review board and human research ethics committee of Beijing Center for Disease Prevention and Control.

## 3. Results

### 3.1. Influenza Virus Activity

**Figure 1:**
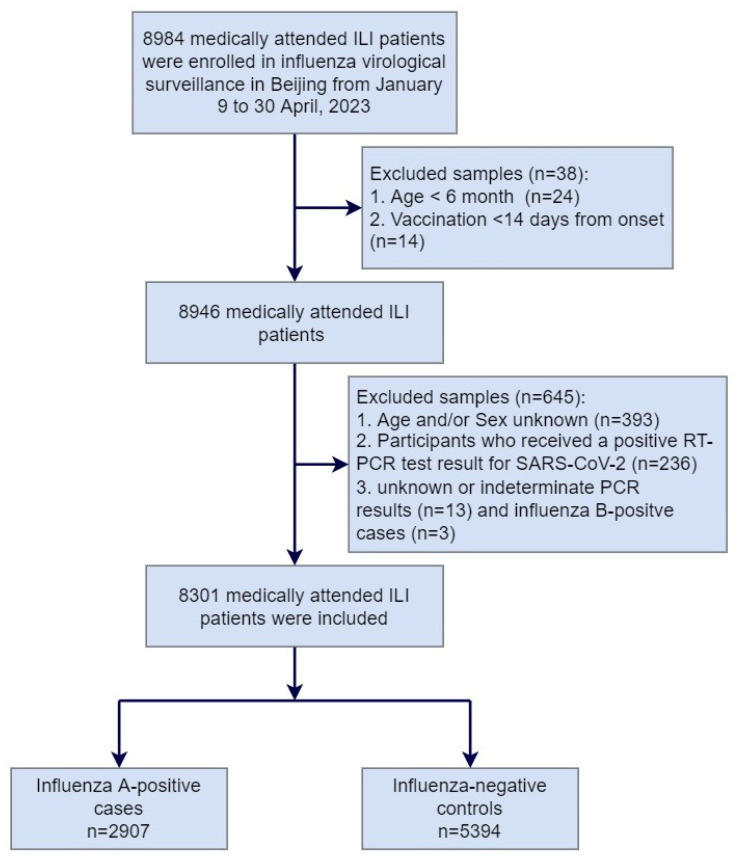
Flow chart of subject enrollment in the test-negative design for estimating influenza vaccine effectiveness in Beijing, China, for the 2022–2023 influenza season.

**Figure 2:**
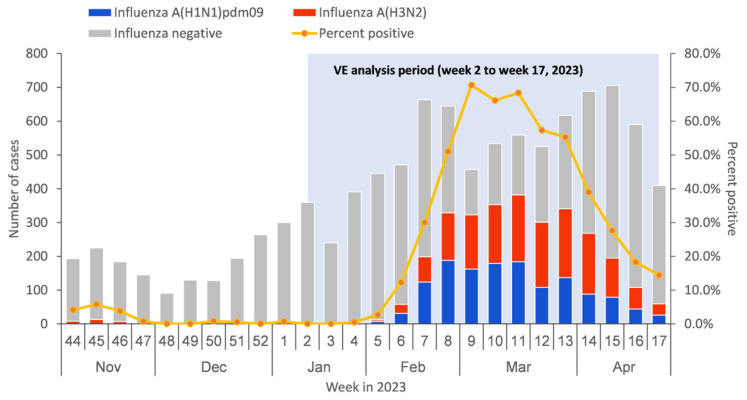
Timeline of recruitment of influenza-like illness (ILI) cases testing positive or negative for influenza virus by type/subtype in Beijing, China, for 1 November 2022–30 April 2023.

From 9 January to 30 April 2023, a total of 8984 medically attended ILI patients were sampled in the sentinel hospitals and were tested in the collaborating laboratories, among which 683 patients were excluded due to not meeting the inclusion criteria or having incomplete surveillance documentation. The specifics of exclusions are shown in Figure 1. Among the 8301 medically attended ILI patients included in the study, 2907 (35.0%) were influenza A-positive cases, and 5394 (65.0%) were influenza-negative controls. Among influenza A-positive cases, 1342 (46.2%) had influenza A (H1N1)pdm09, 1554 (53.4%) had influenza A (H3N2), and 11 (0.4%) had influenza A (H1N1)pdm09 and A (H3N2) co-infection (Table 1). There were no ILI patients detected positive before week 4 of 2023. The positive rate of influenza virus began to rise from week 5 and reached the peak at week 9, with the peak value of 70.7% (Figure 2).

Among the 35 influenza A(H1N1)09pdm sequenced samples, all viruses tested belonged to one of the subclades of A/Victoria/2570/2019 (H1N1)pdm09-like 6B.1A.5a.2 clade, of which 32 (91.4%) belonged to clade 6B.1A.5a.2a, and 3 (8.6%) belonged to clade 6B.1A.5a.2a.1 (Figure S1). Among the 35 influenza A(H3N2) sequenced samples, all viruses tested belonged to one of the subclades of the A/Darwin/9/2021 (H3N2)-like 3C.2a1b.2a.2 clade, with the majority (91.4%, *n* = 32) belonging to the 3C.2a1b.2a.2a.3a.1 subclade (Figure S2).

### 3.2. Participant Characteristics

The median age of influenza A-positive cases and test-negative controls were 29 years old (IQR: 23) and 31 years old (IQR: 31), respectively. ILI patients who tested positive for influenza A were more likely to be 18–59 and 0.5–17 years old compared to ≥60 years of age (38.2% vs. 35.6% vs. 18.5% positive, respectively; *p* < 0.001) and were less likely to have diseases (7.1% vs. 13.8%, respectively; *p* < 0.001). The proportion of influenza-positive cases in males was significantly higher than that in females (36.5% vs. 33.4%, respectively; *p* = 0.004). The positive rate of influenza within a three-day interval from onset to sampling was significantly higher than that within an interval greater than three days (35.7% vs. 17.7%, respectively; *p* < 0.001) (Table 1).

**Figure 3:**
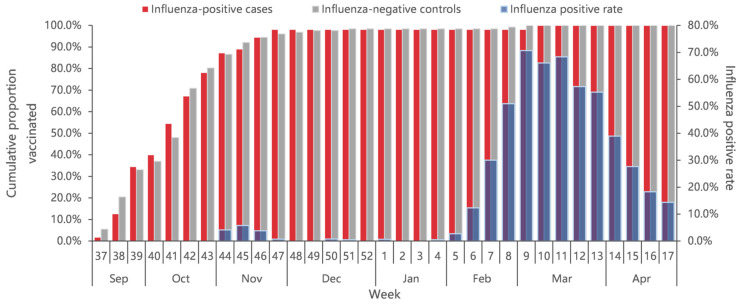
Cumulative proportion of vaccination for influenza-positive cases and influenza-negative controls, and percent positive of influenza by calendar time in Beijing, China, for week 37 of 2022 to week 17 of 2023.

A total of 2.2% ILI patients had received the influenza vaccine (1.9% of 2907 case-patients and 2.4% of 5394 controls), with the overall vaccination rate increasing from early September 2022. Eighty percent of all vaccinee were vaccinated before November 2022, about three months earlier than the start of the influenza epidemic (Figure 3). The vaccination rates among older adults aged ≥60 years and children aged 0.5–17 years were significantly higher than that among 18–59 years of age (*p* < 0.001). The vaccination rate did not differ significantly by sex (*p* = 0.726), presence of chronic diseases (*p* = 0.182), and interval onset to enrollment (*p* = 0.830) (Table 1).

### 3.3. Influenza Vaccination Effectiveness

**Figure 4:**
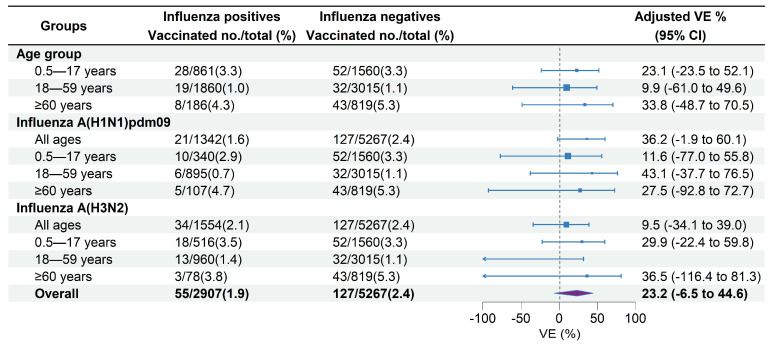
Estimated vaccine effectiveness against influenza A-associated medically attended patients in Beijing, China, for the 2022–2023 influenza season. Abbreviations: CI, confidence interval; VE, vaccine effectiveness.

Among the medically attended ILI patients included, 35.1% of unvaccinated patients versus 32.0% of vaccinated patients tested positive for influenza A (Table 1). The VE against influenza A-associated ILI patients was 23.2% (95% CI: −6.5% to 44.6%) overall across all age groups. The age-specific overall VE was 23.1% (95% CI: −23.5% to 52.1%) in children aged 0.5–17 years, 9.9% (95% CI: −61.0% to 49.6%) in adults aged 18–59 years, and 33.8% (95% CI: −48.7% to 70.5%) in adults aged ≥60 years (Figure 4). VE against influenza A of sensitivity analysis including participants with an interval between ILI onset and swabbing ≤3 days was 22.9% (95% CI: −7.3% to 27.0%), which was similar to the estimate of the primary analysis.

VE against influenza A (H1N1)pdm09 was 36.2% (95% CI: −1.9% to 60.1%) across all age groups, 11.6% (95% CI: −77.0% to 55.8%) in children aged 0.5–17 years, 43.1% (95% CI: −37.7% to 76.5%) in adults aged 18–59 years, and 27.5% (95% CI: −92.8% to 72.7%) in adults aged ≥60 years (Figure 4). VE against influenza A (H3N2) was 9.5% (95% CI: −34.1% to 39.0%) across all age groups, 29.9% (95% CI: −22.4% to 59.8%) in children aged 0.5–17 years, and 36.5% (95% CI: −116.4% to 81.3%) in adults aged ≥60 years. No significant effectiveness against influenza A (H3N2) was observed in adults aged 18–59 years (−32.4%, 95% CI: −157.3% to 31.9%) (Figure 4).

**Figure 5:**
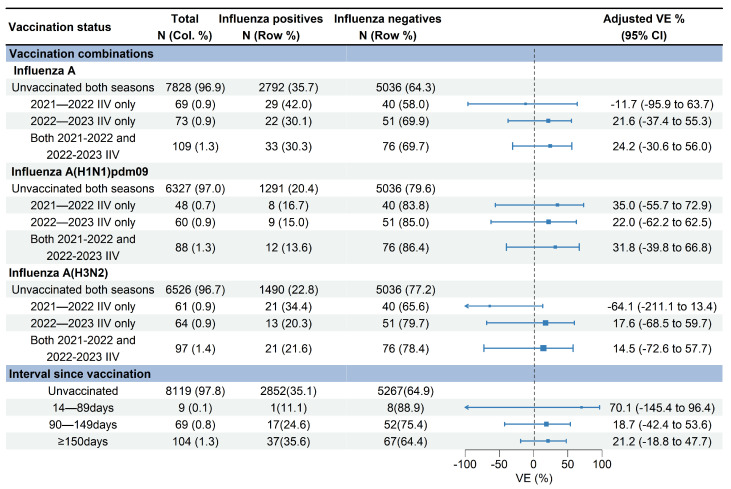
Estimated vaccine effectiveness against influenza A-associated medically attended patients with different vaccination statuses in Beijing, China, for the 2022–2023 influenza season. Abbreviations: CI, confidence interval; VE, vaccine effectiveness; IIV, inactivated influenza vaccine.

VE against influenza A (H1N1)pdm09 for receipt of the 2021–2022 vaccination only, 2022–2023 vaccination only, and vaccinations in both seasons were 35.0% (95% CI: −55.7% to 72.9%), 22.0% (95% CI: −62.2% to 62.5%), and 31.8% (95% CI: −39.8% to 66.8%), respectively (Figure 5). VE against influenza A (H3N2) for receipt of the 2022–2023 vaccination only and vaccinations in both 2021–2022 and 2022–2023 seasons were 17.6% (95% CI: −68.5% to 59.7%) and 14.5% (95% CI: −72.6% to 57.7%), respectively. No significant effectiveness against influenza A (H3N2) was observed for receipt of the 2021–2022 vaccination only (−64.1%, 95% CI: −211.1% to 13.4%) (Figure 5).

### 3.4. VE Changes by Time Intervals between Vaccination and Illness Onset

The median days since vaccination to illness onset was 156 days (IQR: 52) (Figure 3). There was no significant difference of vaccination intervals between influenza positives and test negatives (median days: 158 vs. 155; *p* = 0.628). Time intervals between vaccination and illness onset in children aged 0.5–17 years tended to be longer compared with adults aged 18–59 years and adults aged ≥60 years (median days: 163 vs. 147 vs. 138; *p* = 0.027). We compared VE against influenza A by three defined intervals since vaccination to examine whether the waning of VE was affected by the timing of vaccination. VE of the group with vaccination intervals of 14–90 days was 70.1% (95% CI: −145.4 to 96.4), much higher than that of the group with a vaccination interval of 90–149 days (18.7%, 95% CI: −42.4% to 53.6%) and the group with a vaccination interval of ≥150 days (21.2%, 95% CI: −18.8% to 47.7%) (Figure 5).

## 4. Discussion

After a two-year break of influenza virus circulation at the local and global levels caused by COVID-19-related restrictions from early 2020 [17], the epidemic of influenza returned in succession in most of the countries during the 2021–2022 influenza season, with the predominant of A(H3N2) virus [18]. However, it was not until 2023 that the first influenza epidemic dominated by the influenza A virus after COVID-19 occurred. During the 2022–2023 influenza season, a late-season resurgence of the influenza virus occurred with a record-high level of epidemic intensity in Beijing. This spring wave spanned February to April 2023, with the dominant strains being influenza A(H1N1)pdm09 and A(H3N2) viruses. Influenza vaccination of this season provided modest protection of 23.2% against influenza A-associated medically attended ILI patients. Although the relatively wide confidence intervals around our VE estimates cannot rule out interpretation of no protection, point estimates of VE were still considered the most likely findings [19]. The VE against influenza A(H1N1)pdm09 (36.2%) among all ages was better than that against influenza A(H3N2) (9.5%). The results of age-specific analysis showed that VE point estimates against influenza A(H3N2) among those aged 0.5–17 years and aged ≥60 years were higher than those against influenza A(H1N1)pdm09. VE point estimate among those aged 18–59 years was the highest against influenza A(H1N1)pdm09, while no protective effect was observed against influenza A(H3N2).

Compared with other studies on VE estimation for the 2022–2023 influenza season in the Northern Hemisphere, estimated VE against influenza A and A subtypes in our study was relatively lower. Estimated VE against influenza A-associated emergency department/urgent care (ED/UC) visits was 44% (95% CI: 40% to 47%) overall in the United States (US) [20]. VE against influenza A(H1N1)pdm09 and A(H3N2) among primary care practitioners were 46% (95% CI: 35% to56%) and 36% (95% CI: 25% to 45%) in Europe, respectively [21]. Canada experienced an unusual early 2022–2023 influenza A(H3N2) epidemic, and the vaccine provided a substantial protection with A(H3N2) VE of 54% (95% CI: 38% to 66%) overall [22]. There are relatively few published studies on the VE assessment during the 2022–2023 influenza season in China. A population-based test-negative design study in Shihezi, China, found that the VE of the influenza vaccine against the influenza A virus was 56.3% (95% CI: 13.6% to 73.6%), among which VE against influenza A(H1N1)pdm09 was 61.3% (95% CI: 28.3% to 79.1%), and VE against influenza A(H3N2) was 41.1% (95% CI: −8.8% to 68.3%) [23]. Although a higher estimated VE was reported in this study, the result that VE against influenza A(H1N1)pdm09 was better than that against influenza A(H3N2) was consistent with ours.

Previous studies found that the VE for the influenza vaccine varied widely due to the variations in virus types/subtypes, geographic locations, age categories, the pre-immunity of the population, the vaccine formulation, and antigenic match with circulating viruses, and the influenza vaccination was less effective against influenza A(H3N2) substantially [24,25,26,27]. The low VE against influenza A(H3N2) in Beijing may be partially attributed to the mismatch between circulating and vaccine strains, as the majority of circulating A(H3N2) strains we detected belonged to the 3C.2a1b.2a.2a.3a.1 clade rather than the 3C.2a1b.2a.2 clade of vaccine strains. In addition, egg-based, regular-dose inactivated vaccines were administered during the 2022–2023 influenza season in Beijing, and low response to the egg-based influenza A(H3N2) vaccine strains was reported in China, which may also impact the effectiveness of the vaccine against influenza A(H3N2). Furthermore, the influenza A(H3N2) virus had already been the dominant circulating virus and was widely prevalent in the community in Canada, the US, and Europe during the previous influenza season (2021–2022), which was distinguished from the epidemic in Beijing. Therefore, populations in these countries may have developed a certain level of pre-immunity during this influenza season, and coupled with the fact that the circulating A(H3N2) virus in 2022–2023 was similar to the previous influenza season, VE was generally higher than ours during the 2022–2023 influenza season. However, the extent to which the current VE may be affected by the relative pause in influenza virus circulation during the COVID-19 pandemic is still uncertain. Other factors, such as low vaccination rate and delayed epidemic, may also contribute to the low VE in Beijing.

Our study found that the VE against influenza A-associated medically attended ILI patients was higher in the first three months after receiving the vaccine and reduced significantly after more than three months of vaccination, with VE dropping by about 50% in two time intervals. This finding was consistent with some previous studies reporting a waning VE over time [28,29,30,31]. An estimation of influenza VE among hospitalized children from 2012 to 2017 in Hong Kong, China, found that VE point estimates decreased from 79% for children vaccinated within 0.5 to 2 months to 45% after 6 months of vaccination, dropping by 2 to 5 percentage points per month [7]. Compared with other studies, VE dropped relatively more in our study. Due to the wide confidence interval for an early period since vaccination, which may be caused by the small number of cases, the effectiveness of the influenza vaccine at this stage was somewhat uncertain. In addition, because of the low number of vaccinated cases, the changes in VE for influenza A subtypes and ages were not estimated in our study. A meta-analysis that included 14 TND studies compared VE 15–90 days after vaccination to VE 91–180 days after vaccination, finding a more significant decrease in VE for influenza A(H3N2) than that for influenza A(H1N1)pdm09 (−33% vs. −8% change in VE) [32]. The study also suggests that the change in VE was associated with the proportion of influenza-positive cases and test-negative controls that participated in the study, which could reflect biological effects such as mismatch between the vaccine received and the circulating strains, herd immunity among controls, or the reduced power of individual TND studies in the later parts of an influenza outbreak. Due to the small number of patients, our study did not analyze the change of VE by influenza A subtypes and by age groups, and further research is needed in these aspects in the future.

Several limitations should be considered in the study. First, low vaccination coverage during the 2022–2023 influenza season in Beijing, particularly among adults aged 18–59 years, coupled with the small number of influenza-positive cases vaccinated, may limit statistical power and widen confidence intervals of the results in the study. It should be noted that due to the negative 95%CI of VE estimates in certain subgroups, there are limitations to the generalizability of the results. It was indicated that results of VE estimation are most likely to be certain when the vaccine coverage is between 20 and 80% [33]. Second, healthcare-seeking behavior may be changed by the wave of the COVID-19 epidemic in early 2023 in Beijing, and enrollment of medically attended patients might have had an uncertain impact on the results. Third, the VE estimated in this study was specific to the prevention of outpatient influenza patients, and it may not represent the VE for more severe influenza outcomes. Fourth, VE was estimated based on a proportion of the ILI patients seeking medical care. The care-seeking behavior of people can be influenced by many factors, such as the vaccination status, the severity of the symptoms, the time of the year, and so on. Therefore, bias may be introduced. Fifth, because the study did not involve mild and asymptomatic infections, VE may be overestimated when compared to its effectiveness in the general population that were not hospitalized. Finally, there may be other residual biases, and confounding cannot be ruled out.

## 5. Conclusions

In conclusion, during the delayed 2022–2023 influenza season in Beijing, we observed a modest VE against influenza A-associated medically attended ILI visits, with a better VE against influenza A(H1N1)pdm09 and a low VE against influenza A(H3N2). Estimated VE decreased significantly 3 months after vaccination, indicating a potential need to optimize current influenza vaccination policy, such as providing a second influenza vaccination 3 months after the initial vaccination campaign each influenza season, especially for vulnerable populations. To address the issue of the inadequate effectiveness of influenza vaccine caused by vaccine strain mismatch and vaccination timing, it is crucial to enhance continuous monitoring of influenza epidemic trends, viral antigenic and genetic characteristics, and analysis of viral evolution patterns. By timely tracking the epidemiological and pathogenic characteristics of influenza, public health officials can make more informed decisions regarding vaccine formulation and the optimal timing of vaccination for future seasons in Beijing, providing better protection by influenza vaccination.

## Figures and Tables

**Table 1:** Characteristics of influenza-positive cases and test-negative controls, as well as influenza vaccination status of the participants included in the influenza vaccine effectiveness assessment in Beijing, China, for the 2022–2023 influenza season.

Characteristics	Total No. (Col. %)	Influenza Test Result				Influenza Vaccination Status		
		All Influenza A Cases No. (Row %)	Influenza A(H1N1) pdm09 Cases No. (Row %)	Influenza A(H3N2) Cases No. (Row %)	Test-Negative Controls No. (Row %)	Unvaccinated No. (Row %)	Vaccinated No. (Row %)	*p* Value
**Total**	**8301 (100.0)**	**2907 (35.0)**	**1342 (16.1)**	**1554 (18.7)**	**5394 (65.0)**	**8119 (97.8)**	**182 (2.2)**	
**Sex**								
Female	3930 (47.3)	1313 (33.4)	599 (15.2)	710 (18.1)	2617 (66.6)	3841 (97.7)	89 (2.3)	0.726
Male	4371 (52.7)	1594 (36.5)	743 (17.0)	844 (19.3)	2777 (63.5)	4278 (97.9)	93 (2.1)	
**Age group**								
0.5–17 years	2421 (29.2)	861 (35.6)	340 (14.0)	516 (21.6)	1560 (64.4)	2341 (96.7)	80 (3.3)	<0.001
18–59 years	4875 (58.7)	1860 (38.2)	895 (18.4)	960 (19.8)	3015 (62.1)	4824 (99.0)	51 (1.0)	
≥60 years	1005 (12.1)	186 (18.5)	107 (10.6)	78 (7.8)	819 (81.5)	954 (94.9)	51 (5.1)	
**Any chronic condition**								
No	7351 (88.6)	2702 (36.8)	1222 (16.6)	1470 (20.0)	4649 (63.2)	7196 (97.9)	155 (2.1)	0.182
Yes	950 (11.4)	205 (21.6)	120 (12.6)	84 (8.8)	745 (78.4)	923 (97.2)	27 (2.8)	
**Month of ILI symptom onset**								
January 2023	1144 (13.8)	6 (0.5)	6 (0.5)	0 (0)	1138 (99.5)	1119 (97.8)	25 (2.2)	<0.001
February 2023	2239 (27.0)	714 (31.9)	413 (18.4)	300 (13.4)	1525 (68.1)	2205 (98.5)	34 (1.5)	
March 2023	2258 (27.2)	1413 (62.6)	632 (28.0)	773 (34.2)	845 (37.4)	2216 (98.1)	42 (1.9)	
April 2023	2660 (32.2)	774 (29.1)	291 (10.9)	481 (18.1)	1886 (70.9)	2579 (97.0)	81 (3.0)	
**Time interval between ILI onset and swabbing**								
0–3 days	7984 (96.2)	2851 (35.7)	1317 (16.5)	1523 (19.1)	5133 (64.3)	7810 (97.8)	174 (2.2)	0.830
≥4 days	317 (3.8)	56 (17.7)	25 (7.9)	31 (9.8)	261 (82.3)	309 (97.5)	8 (2.5)	
**Influenza vacciation status**								
Unvaccinated	8119 (97.8)	2852 (35.1)	1321(16.3)	1520 (18.7)	5267 (64.9)	8119 (100.0)	0 (0.0)	
Vaccinated	182 (2.2)	55 (30.2)	21 (11.5)	34 (18.7)	127 (69.8)	0 (0.0)	182 (100.0)	

## Data Availability

The original database containing confidential patient information cannot be made publicly available. The anonymized data used in this study are available based on reasonable request to the corresponding author.

## Supplementary Materials

The following supporting information can be downloaded at: https://www.mdpi.com/article/10.3390/vaccines12101124/s1, Figure S1: Phylogentic analysis of haemagglutinin (HA) genes of influenza A(H1N1)pdm09 viruses from February to April 2023 in Beijing, China; Figure S2: Phylogentic analysis of haemagglutinin (HA) genes of influenza A(H3N2) viruses from February to April 2023 in Beijing, China.
