# Supplementary material for: Influenza Vaccine Effectiveness against Influenza A-Associated Outpatient and Emergency-Department-Attended Influenza-like Illness during the Delayed 2022–2023 Season in Beijing, China
Source: Vaccines (Basel). 2024 Sep 30;12(10):1124. doi: 10.3390/vaccines12101124 (PMC11511380; doi:10.3390/vaccines12101124)
Supplement: Supplementary file 1 [file vaccines-12-01124-s001.zip › vaccines-3193611-supplementary.pdf]

# Influenza Vaccine Effectiveness against Influenza A-Associated Outpatient and Emergency-Department-Attended Influenza-Like Illness during the Delayed 2022–2023 Season in Beijing, China

**Li Zhang [1], Guilan Lu [1], Chunna Ma [1], Jiaojiao Zhang [1], Jia Li [1], Wei Duan [1], Jiaxin Ma [1], Weixian Shi [1], Yingying Wang [1], Ying Sun [1], Daitao Zhang [1,2], Quanyi Wang [1,2] and Da Huo [1,3*]**

[1] Institute for Infectious Disease and Endemic Disease Control, Beijing Center for Disease Prevention and Control, No.16 He Pingli Middle St, Dongcheng District, Beijing 100013, China
[2] Beijing Research Center for Respiratory Infectious Diseases, No.16 He Pingli Middle St, Dongcheng District, Beijing 100013, China
[3] School of Public Health, Capital Medical University, 10 Xitoutiao You'anmenwai St, Fengtai District, Beijing 100069, China
[*]Correspondence: Author: Da Huo, Email: huoda@bjcdc.org

# Supplemental Figures

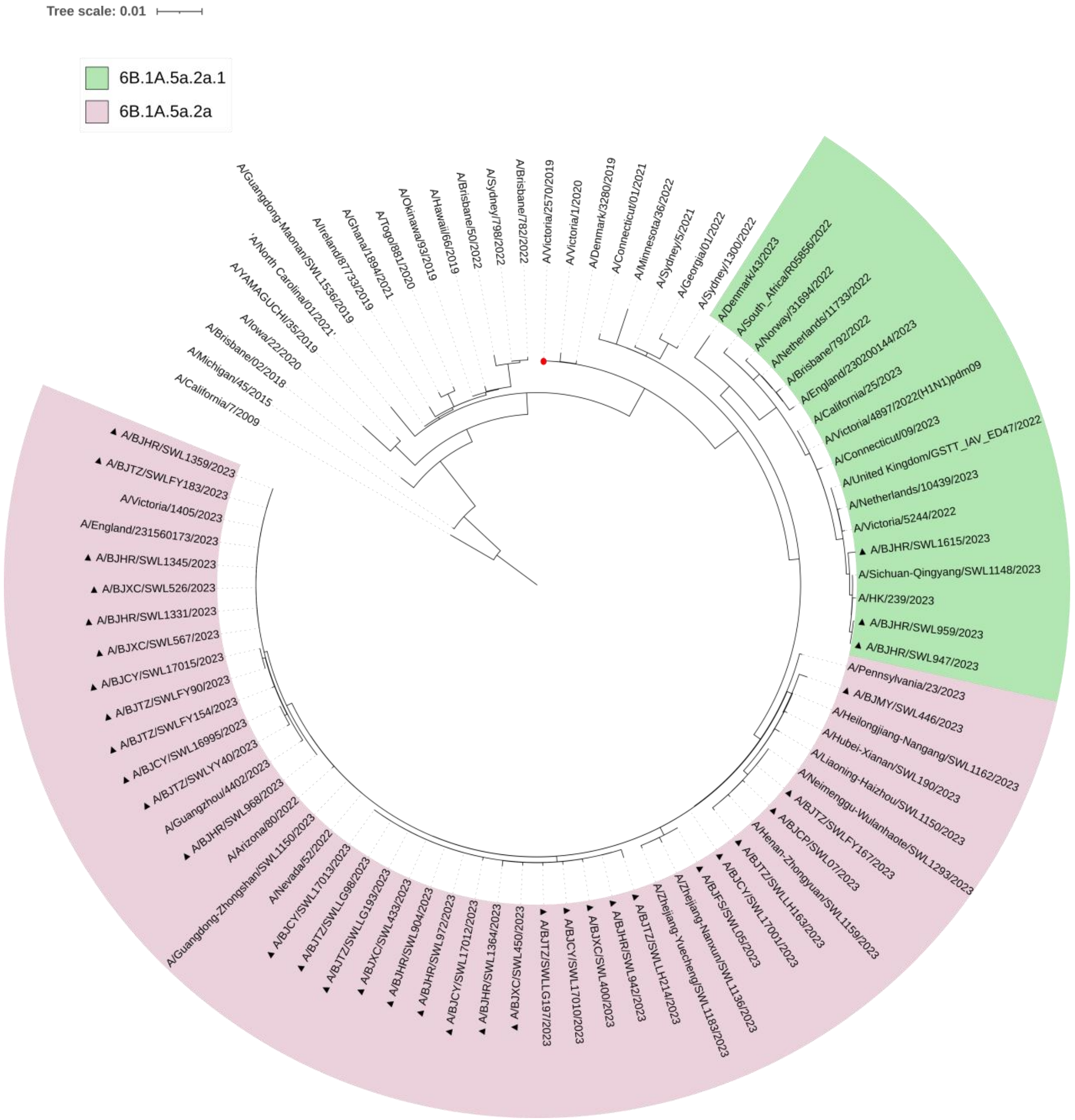


**Figure S1.** Phylogentic analysis of haemagglutinin (HA) genes of influenza A(H1N1)pdm09 viruses from February to April, 2023 in Beijing, China. Circulating influenza viruses analyzed for the purpose of this study are indicated by black solid triangles, while vaccine strains are represented by red solid dot.

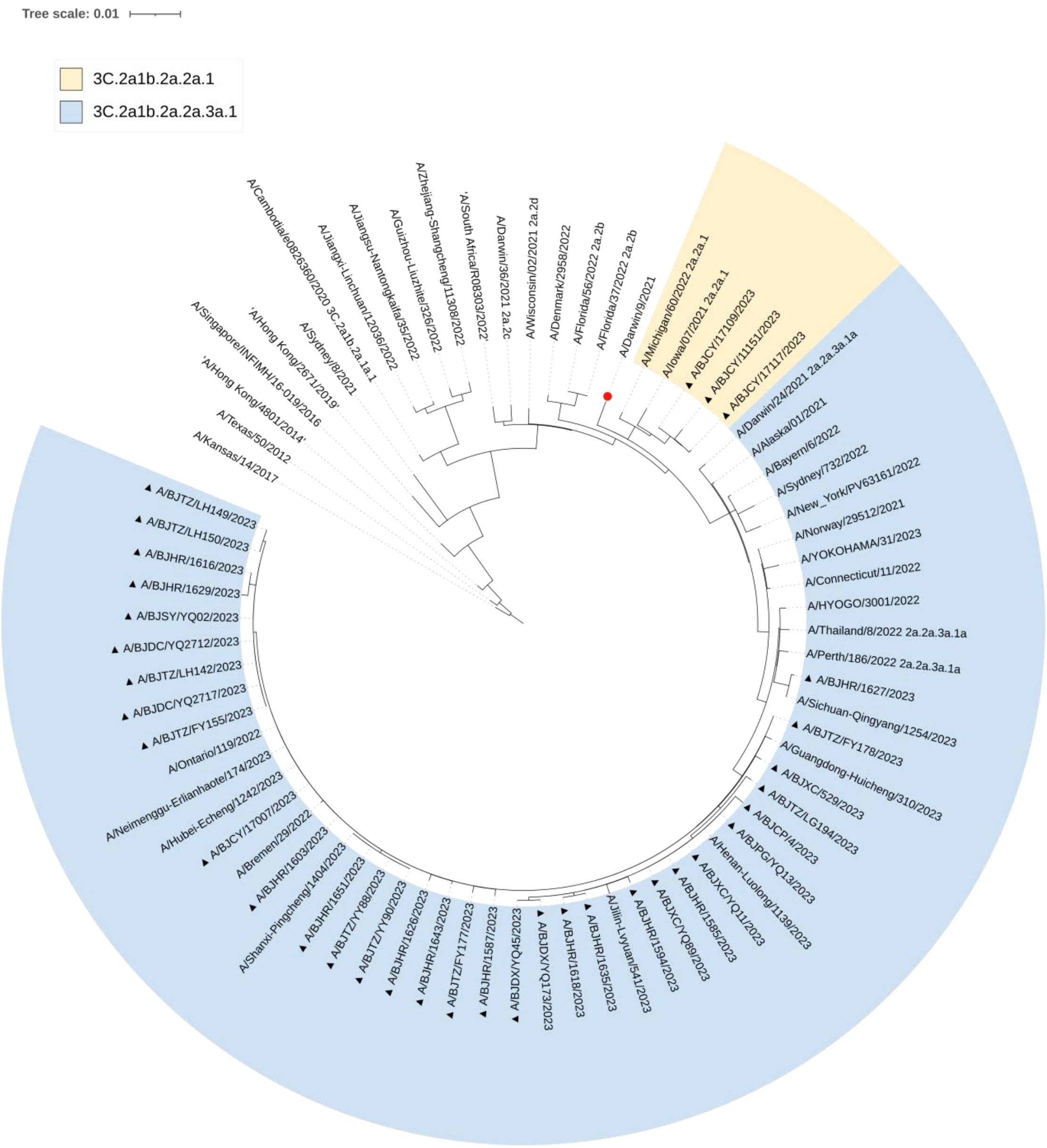


**Figure S2.** Phylogentic analysis of haemagglutinin (HA) genes of influenza A(H3N2) viruses from February to April, 2023 in Beijing, China. Circulating influenza viruses analyzed for the purpose of this study are indicated by black solid triangles, while vaccine strains are represented by red solid dot.
